# Acute Generalized Exanthematous Pustulosis Secondary to Valproic Acid and Risperidone in a Patient with Bipolar I Disorder with Psychotic Features: A Case Report

**DOI:** 10.1192/j.eurpsy.2026.11505

**Published:** 2026-07-31

**Authors:** I. C. C. Preclaro, I. C. Tan, R. Paccial, I. A. C. Preclaro

**Affiliations:** 1 Mariveles Mental Wellness Ans General Hospital, Bataan; 2 Dermatology And Venereology, Tondo Medical Center, Manila; 3 molecular Medicine - College Of Medicine, St. Luke’s Medical Center, Quezon City, Philippines

## Abstract

**Introduction:**

Acute Generalized Exanthematous Pustulosis (AGEP) is an uncommon drug eruption reaction following intake of medications such as antibiotic, antihypertensive, and antiepileptic drugs. When untreated, this can lead to severe organ dysfunction. With the use of psychotropic and antiepileptic medications in the treatment of bipolar disorder, existing published literature about AGEP in patients with bipolar disorder treated with psychotropics and antiepileptic medications remains relatively rare. Managing AGEP involves supportive care, corticosteroid and antihistamine therapy.

**Objectives:**

To describe a case of acute generalized exanthematous pustulosis in a patient with bipolar disorder, highlighting the clinical presentation, diagnostic process in resource-limited setting, and management.

**Methods:**

This case report aims to describe the course of treatment of a Filipino woman diagnosed with bipolar disorder with psychosis who developed acute generalized exanthematous pustulosis while being treated with Risperidone and Valproic acid.

**Results:**

A 57-year-old female was admitted at the Psychiatric unit as a case of Bipolar I disorder, current episode manic with psychotic features. She was managed with Olanzapine 10mg/tab per day and Valproic Acid 1000mg/tab per day. She was initially noncompliant with Valproic acid and preferred Olanzapine monotherapy. After a few days, the Olanzapine dose was increased to 20 mg per day due to persistent psychosis. However, since there was no improvement in psychosis by hospital day 9, the medication was switched to Risperidone 2mg per day. On hospital day 10, with regular intake of Risperidone and Valproic acid, she developed erythematous papular lesions on the anterior and posterior trunk, which progressed to generalized erythematous pustular lesions involving the neck, trunk, upper extremities, thighs, and the groin, sparing the oral mucosa, palms, soles, and feet. Febrile episodes (37.9 to 39.1C) were noted. Complete blood count revealed neutrophilia (18,500/mm3) and a decrease in platelet count (132,000/mm3). Both medications were immediately discontinued. Referral to Dermatology was done and was diagnosed clinically, with Acute Generalized Exanthematous Pustulosis secondary to use of Risperidone and Valproic acid (EuroSCAR 9). She was started on intravenous and topical corticosteroids, leading to improvement of her lesions after eight days of continuous treatment.

**Conclusions:**

Vigilant monitoring is necessary whenever a patient starts with psychotropic medications because side effects like AGEP may occur within the first week of treatment. Early initiation of intravenous and cutaneous treatment can provide rapid relief of symptoms.

**Disclosure of Interest:**

None Declared

